# AMEEGNet: attention-based multiscale EEGNet for effective motor imagery EEG decoding

**DOI:** 10.3389/fnbot.2025.1540033

**Published:** 2025-01-22

**Authors:** Xuejian Wu, Yaqi Chu, Qing Li, Yang Luo, Yiwen Zhao, Xingang Zhao

**Affiliations:** ^1^State Key Laboratory of Robotics, Shenyang Institute of Automation, Chinese Academy of Sciences, Shenyang, China; ^2^University of Chinese Academy of Sciences, Beijing, China; ^3^School of Information Science and Engineering, Shenyang University of Chemical Technology, Shenyang, China

**Keywords:** motor imagery (MI) EEG, brain-computer interface, signal decoding, multi-scale decoding, fusion transmission, efficient channel attention (ECA) mechanism

## Abstract

Recently, electroencephalogram (EEG) based on motor imagery (MI) have gained significant traction in brain-computer interface (BCI) technology, particularly for the rehabilitation of paralyzed patients. But the low signal-to-noise ratio of MI EEG makes it difficult to decode effectively and hinders the development of BCI. In this paper, a method of attention-based multiscale EEGNet (AMEEGNet) was proposed to improve the decoding performance of MI-EEG. First, three parallel EEGNets with fusion transmission method were employed to extract the high-quality temporal-spatial feature of EEG data from multiple scales. Then, the efficient channel attention (ECA) module enhances the acquisition of more discriminative spatial features through a lightweight approach that weights critical channels. The experimental results demonstrated that the proposed model achieves decoding accuracies of 81.17, 89.83, and 95.49% on BCI-2a, 2b and HGD datasets. The results show that the proposed AMEEGNet effectively decodes temporal-spatial features, providing a novel perspective on MI-EEG decoding and advancing future BCI applications.

## 1 Introduction

Brain-computer interface (BCI) is an innovative interdisciplinary research field that combines biomedical science, neuroscience, and human-computer interaction. Its aim is to establish a direct bidirectional communication channel between the brain and external devices, such as robotic arms and humanoid robots, bypassing the peripheral nerves and thereby enhancing the quality of life for individuals with disabilities (Wolpaw et al., 2020; Mane et al., 2020; Robinson et al., 2021).

Compared to evoked electroencephalography (EEG) (Gefferie et al., 2023), motor imagery (MI) EEG is inherently spontaneous, which provides unique advantages in BCI technology by enabling control without external environmental interference (Sharma et al., 2023). In the process of collecting MI EEG signals, researchers have found that different motor imagery tasks activate distinct brain regions (Eaves et al., 2024). During both ipsilateral and contralateral movements, electrical signals from the sensorimotor cortex exhibit varying amplitude responses in the frequency bands of *α* (8–12) Hz and *β* (13–30) Hz, known as event-related synchronization (ERS) and event-related desynchronization (ERD) respectively (Tariq et al., 2020). Leveraging these phenomena, researchers have developed various feature extraction methods in the temporal, frequency, and spatial domains, including short-time Fourier transform (STFT), continuous wavelet transform (CWT), and common spatial pattern (CSP), along with other variant algorithms (Shayeste and Asl, 2023; Zhang et al., 2020; You et al., 2020). Additionally, a variety of classification algorithms such as artificial neural networks (ANN), support vector machines (SVM), and Bayesian classifiers have been employed (Zhang et al., 2019; Luo et al., 2020; Tortora et al., 2020). These traditional machine learning methods have the poor performance for EEG decoding due to their limited feature extraction capability. For example, while CSP focuses on extracting spatial domain features, it is influenced by noise and artifacts, which can obscure important information. In addition, it also overlooks the temporal features of EEG signals.

In recent years, deep learning has played a significant role in improving EEG-based MI-BCI. Among these deep learning architectures, convolutional neural networks (CNN) are widely used for their ability to learn features from EEG datasets, demonstrating strong performance in the prediction and classification of various EEG signal (Schirrmeister et al., 2017; Ozcan and Erturk, 2019; Wang et al., 2019). For instance, Liu et al. (2023) proposed an end-to-end CNN model named SincMSNet, which extracts features through spatial convolutions and temporal logarithmic variance. Lawhern et al. (2018) introduced EEGNet, a deep learning framework adaptable to various EEG signals. This framework combines deep and shallow convolutional structures, enabling training on limited datasets while achieving high decoding accuracy and reduced training time, thereby enhancing model efficiency and generalization capability, making it an ideal tool for processing EEG signals. Some researchers use multi-scale method to learn EEG features. Liu et al. (2022) proposed a network designed to derive a multi-scale spectral representation of EEG data. Zhang et al. (2021) employed the EEG-inception method to extract multi-scale EEG features through augmentation techniques. Amin et al. (2021) utilized different convolution kernels for multi-scale EEG feature extraction and incorporated BiLSTM to enhance the modeling effectiveness in the time domain. While these methods leverage various convolution kernels for multi-scale feature extraction, they face limitations in the number of scales extracted. Adding more scales can significantly increase the number of parameters, resulting in models that are not lightweight.

Models based on attention mechanisms have gained popularity in EEG decoding due to their ability to selectively process specific information while filtering out irrelevant data. Many researchers have integrated attention mechanisms into deep learning to focus on important decoding information, thereby enhancing MI-EEG decoding performance. Xie et al. (2022) proposed a deep learning structure based on the multi-head self-attention mechanism for EEG classification. Additionally, Altaheri et al. (2022) combined attention mechanisms with temporal convolutional networks to emphasize the most valuable features. These methods demonstrate better performance with attention mechanisms, highlighting that focusing on important EEG signals is an effective way to enhance the accuracy and robustness of EEG decoding.

In light of the above, to enhance the robustness and decoding performance of MI-EEG decoding, an attention-based multiscale EEGNet (AMEEGNet) for effective MI-EEG decoding is proposed. This model effectively decodes MI EEG across multiple scales and further increases the decoding scales through fusion transmission, all while introducing only a minimal number of additional parameters. The lightweight ECA mechanism significantly improves performance without incurring substantial overhead. AMEEGNet enhances the decoding capabilities of EEGNet and can be seamlessly integrated into networks that utilize EEGNet, serving as a replacement to elevate overall decoding performance. This article highlights four main contributions and innovations as follows:

1) To address the issue of low performance in temporal-spatial EEG decoding, a multi-scale EEGNet is utilized to extract features across various scales, enabling the model to learn temporal-spatial features from different EEG domain. This approach significantly boosts the extraction capabilities of the model, allowing it to capture both temporal and spatial features at multiple scales. As a result, it deepens the understanding of complex EEG signals and improves the feature extraction and generalization abilities of the model.
2) To further enhance the multi-scale decoding effect of the model while avoiding complex architecture, fusion transmission is employed to comprehensively analyze EEG signal features across different temporal and spatial scales. Fusion transmission requires parallel networks to facilitate parameter sharing, thereby improving the degree of multiscale interaction. This innovative method significantly expands feature extraction capabilities of the model, providing a more comprehensive representation of EEG activity. As a result, this approach overcomes the limitations of traditional architectural methods and enhances the accuracy of EEG signal decoding.
3) To enhance the model’s ability to focus on important channels while reducing computational burden, the efficient channel attention (ECA) mechanism is employed (Wang et al., 2020). This lightweight approach emphasizes key channels, facilitating the acquisition of more discriminative spatial features. By assigning greater weights to critical EEG signal features, this method avoids complex dimensionality reduction and enhancement processes, achieving efficient and lightweight feature extraction.
4) The model achieved excellent results on the BCI Competition IV 2a, 2b and HGD datasets. Additionally, visualization techniques were employed to analyze the model, enhancing interpretability and comprehensive validation of the proposed method’s performance.

## 2 Methods

### 2.1 Motor imagery datasets

BCI Competition IV 2a dataset is a key resource for research in MI-EEG decoding, including four MI classification tasks. It includes EEG recordings from 22 electrodes positioned on nine participants (Brunner et al., 2008), sampled at 250 Hz and filtered within the range of 0.5 Hz to 100 Hz. The four MI classification tasks involve: left hand, right hand, foot, and tongue. Each participant completed two sessions on separate days, with each session comprising six runs, and each run containing 48 trials. One of these sessions is designated for model training, while the other serves for evaluation.

BCI Competition IV 2b dataset is designed for two specific classification tasks and contains EEG signals collected from three electrodes positioned on nine participants (Leeb et al., 2008). It shares the same sampling frequency of 250 Hz and filtering range of 0.5 Hz to 100 Hz. The two MI classification tasks focus on left hand and right hand. Each subject engaged in five sessions; the first two sessions include 120 trials each, while the last three sessions comprise 160 trials each. The initial three sessions are utilized for training the model, while the final two sessions are reserved for evaluation.

The High Gamma Dataset (HGD) is designed for a four-class MI classification task (Schirrmeister et al., 2017). It includes EEG recordings from 44 electrodes positioned on 14 healthy subjects, with each subject participating in 13 runs. The four MI classification tasks consist of left hand, right hand, foot, and rest (no movement). For each subject, the training set comprises approximately 880 trials (all runs except the last two), while the test set includes around 160 trials (the last two runs). The original sampling rate for HGD is 500 Hz; however, for consistency with similar research involving BCI Competition IV 2a and 2b, the dataset was resampled to 250 Hz.

### 2.2 Data preprocessing

**Figure 1:**
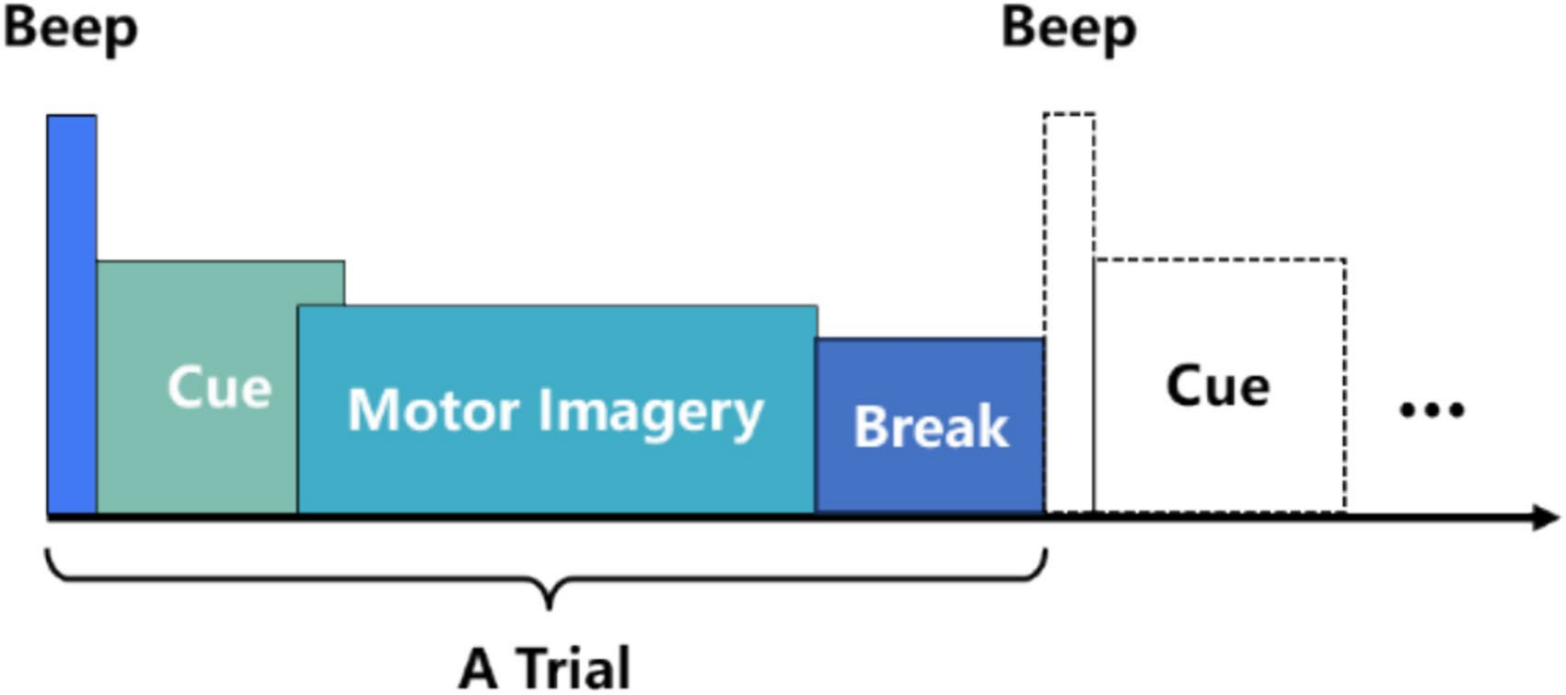
A timing scheme for a motor imagery EEG acquisition experiment.

In MI-EEG decoding, data preprocessing is typically employed to remove noise and artifacts generated during experiments, thereby enhancing signal quality and ensuring the accuracy of MI-EEG decoding. However, this process can lead to data loss. AMEEGNet is an end-to-end lightweight network that does not require EEG noise or artifact removal, nor bandpass filtering, the only preprocessing method employed is data segmentation. This approach allows AMEEGNet model to learn relevant signal features directly from the raw data, avoiding potential data loss associated with preprocessing methods. Figure 1 illustrates the timing scheme for a motor imagery EEG (MI-EEG) acquisition experiment. Data segmentation focuses on the “cue” and “motor imagery” phases, which are critical for capturing signals from the brain (Sharma et al., 2023). According to the collection processes of datasets (Schirrmeister et al., 2017; Brunner et al., 2008; Leeb et al., 2008; Ohyver et al., 2019), the “cue” and “motor imagery” phases are segmented as follows: the BCI IV 2a dataset is segmented over the time interval [1.5 s, 6 s], resulting in 1,125 samples; the BCI IV 2b dataset is segmented over the time interval [0 s, 4 s], resulting in 1,000 samples; and the HGD dataset is also segmented over the time interval [0 s, 4 s], yielding 1,000 samples.

### 2.3 Overall structure of AMEEGNet

To enhance feature extraction performance and improve the ability to focus on important features for motor imagery EEG (MI-EEG) decoding, thereby increasing robustness and decoding accuracy of model, an attention-based multiscale EEGNet (AMEEGNet) is proposed. This framework consists of three key blocks for MI-EEG decoding: block 1 is parallel EEGNet block; block 2 is efficient channel attention block; block 3 is classification block.

**Figure 2:**
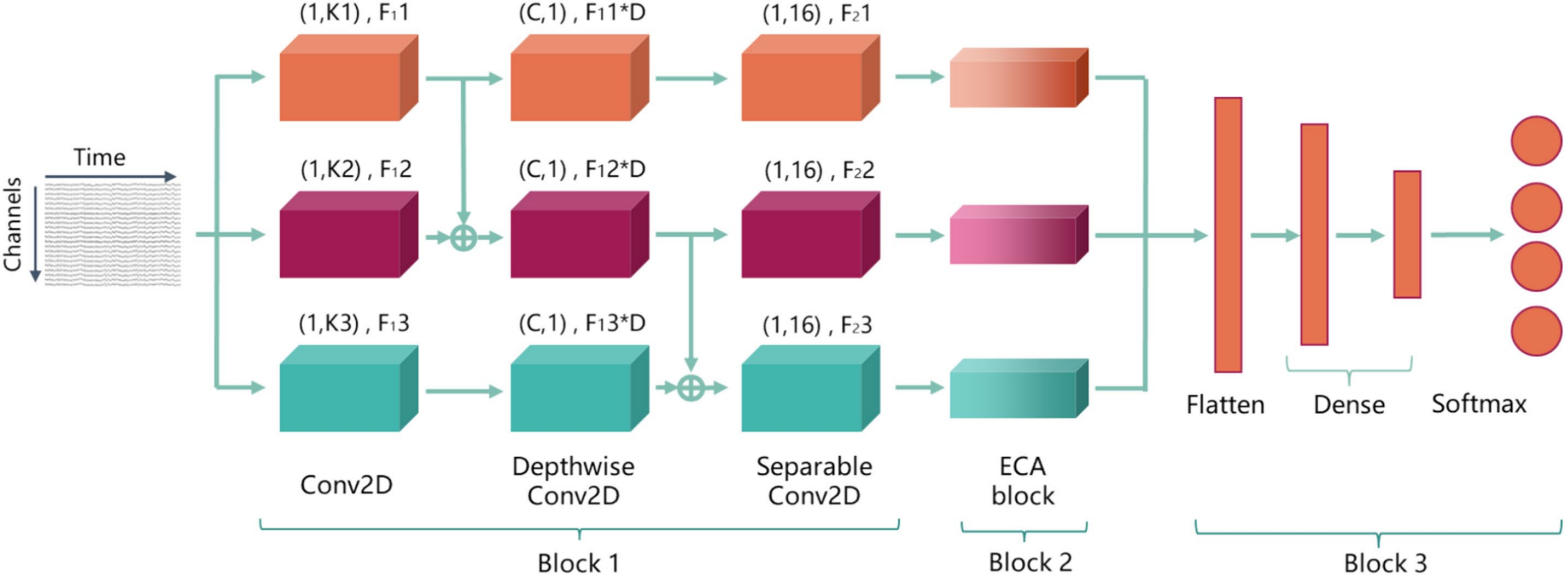
The overall structure of AMEEGNet, including three blocks: block 1 is parallel EEGNet block; block 2 is efficient channel attention block; block 3 is classification block.

In parallel EEGNet block, raw EEG data is direct input into three parallel EEGNet architectures, each with different parameters, allowing for the extraction of temporal-spatial features from raw EEG signals at multiple scales; the ECA block is a lightweight attention mechanism that assigns varying weights to different channels, optimizing network parameters adaptively based on feature importance; the classification block includes a flatten layer followed by two dense layers. Finally, the softmax activation function is used to classify MI-EEG tasks. Figure 2 is the overall structure of AMEEGNet.

#### 2.3.1 Parallel EEGNet block

In this work, the block consists of three parallel EEGNet architectures, each extracting temporal-spatial features from EEG data at multiple scales. All three EEGNets process the same temporal-domain and spatial-domain EEG signals. The input of them is a two-dimensional EEG signal. EEGNet includes three convolutional layers: the first layer is a 2D convolutional layer that enables the model to flexibly extract features relevant to the decoding task. The second layer is a depthwise convolutional layer that allows the model to learn features independently for each channel, effectively capturing the complex relationships between different channels and enhancing decoding accuracy. The third layer is a separable convolutional layer that helps the model extract more representative features through the combination of various attributes, enabling it to learn rich features with fewer parameters.

The first layer performs a temporal convolution with *F*_1_*i* filters of size (1, *Ki*), where the *i* = 1, 2, 3, represents the three parallel EEGNet architectures, and *Ki* indicates the filter length for EEGNet *i* along the time axis. *F*_1_*i* represents the temporal feature maps output by this layer of EEGNet *i*. Specifically, *F*_1_1 = 4 and *K*1 = 16; and *F*_1_2 = 2 × *F*_1_1, *F*_1_3 = 2 × *F*_1_2, *K*2 = 2 × *K*1, and *K*3 = 2 × *K*2. It can extract EEG signal features on three time scales and obtain more feature maps in those time scales. The depthwise convolutional layer with *F*_1_*i* × *D* filters of size (*C*, 1), where the *C* is the number of EEG channels and the *D* is the number of filters associated with each temporal feature map from the previous layer, empirically set to 2. The final layers utilize *F*_2_*i* filters of size (1, 16), where the *F*_2_*i* = *F*_1_*i* × *D*. These varying parameters enable the extraction of different lengths of temporal features and multi-dimensional spatial features, resulting in effective multi-scale feature extraction. Batch normalization (BN) and activation layers of exponential linear unit (ELU) are added after the second and third layers to normalize outputs and introduce non-linearity. This enhances model stability and improves the learning of complex patterns in the EEG signals.

More importantly, although parallel architectures can enhance the effectiveness of multi-scale feature extraction, they also have limited effect in feature extraction. Continuing to increase parallel architectures may lead to gradient disappearance or explosion, raising the risk of overfitting. Therefore, to improve multi-scale feature extraction performance while avoiding complex model architectures, fusion transmission is employed to comprehensively analyze EEG signal features across various temporal and spatial scales. The output from the 2D convolutional layer of EEGNet 1 is combined with the output from the 2D convolutional layer of EEGNet 2 and fed into the depthwise convolutional layer of EEGNet 2. This allows the depthwise convolutional layer to analyze features with two different filter lengths and temporal feature maps, further facilitating multi-scale feature extraction. Subsequently, the outputs from the depthwise convolutional layers of EEGNet 2 and EEGNet 3 are combined to create a feature that integrates multi-scale information, serving as the input to the separable convolutional layer of EEGNet 3. Fusion transmission enables a deeper integration of multi-scale features, allowing the layers to extract and analyze a broader range of EEG signals. With this method, the three parallel EEGNet architectures put temporal features at different scales into more depthwise and separable convolution layers with varying filters. This approach allows for the learning of more diverse scale features compared to models without fusion transmission, thereby enhancing the effectiveness of multi-scale feature extraction.

#### 2.3.2 Efficient channel attention block

**Figure 3:**
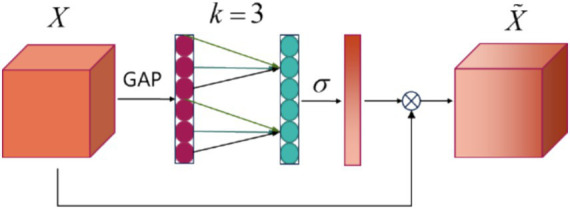
ECA block architecture. *X* is the input feature, GAP represents the Global Average Pooling layer, *k* denotes the size of the 1D convolution, *σ* is the activation function, and $\tilde{X}$ indicates the output data.

In recent studies, inspired by the successful use of attention mechanisms for channel selection in MI-EEG processing as demonstrated in MI-EEG decoding (Liu et al., 2022; Altaheri et al., 2022), a new model has been proposed that utilizes the efficient channel attention (ECA) module for channel selection. The core idea of the ECA module is to capture inter-channel dependencies through 1D convolution. Compared to traditional attention mechanisms, the ECA module bypasses complex dimensionality reduction and expansion processes, resulting in a more efficient and lightweight design. After the depthwise convolutional layer, the output consists of linear combinations of the original channels. This newly formed feature map, treated as a channel, retains valuable information derived from the original EEG signals. The ECA focuses on those feature maps. The architecture of the ECA module is illustrated in Figure 3. The following section will explain the working mechanism of the ECA module and demonstrate how it achieves its objectives.

The output of parallel EEGNet block serves as the input feature *X*, the ECA block applies global average pooling (GAP) across the spatial dimensions to generate a channel descriptor, as shown in Equation 1:


(1) $$z=\mathrm{GAP}\left(X\right)=\frac{1}{H\times W}\overset{H}{\underset{h=1}{\sum }}\overset{W}{\underset{w=1}{\sum }}X$$

The result in the vector *z* of size channels *C*, representing the global information of each channel. And the *k* value, which determines the size of the convolution kernel, is computed based on the number of input channels *C*, the formula for value of *k* is as shown in Equation 2:


(2) $$k=\frac{\log_{2}\left(C\right)+b}{\gamma }$$

where *γ* is a hyperparameter typically set to a constant, and *b* is the bias, they are used to control the kernel size *k*. The value of *k* is usually constrained to small odd numbers, in this block, *k* = 3. Next, a 1D convolution is applied using the computed *k* value to capture channel dependencies, Equation 3 this process of generating convolution output:


(3) $$y=\sigma \left(W*z+b\right)$$

where *W* is the convolution kernel of size *k*, and *σ* is the sigmoid activation function. This step generates an attention score for each channel. Finally, the original feature map *X* is rescaled using the computed channel weights, as shown in Equation 4:


(4) $$\tilde{X}=X⊙y$$

where ⊙ denotes element-wise multiplication, and $\tilde{X}$ is the output feature map with enhanced channel attention. Three output feature maps will be input of classification block.

The ECA block enables the module to focus on important channel contributions, effectively emphasizing significant channels while maintaining a lightweight and computationally efficient structure. This enhancement allows AMEEGNet to achieve improved robustness and effectiveness in MI-EEG decoding.

#### 2.3.3 Classification block

The classification block is used to classify the processed EEG features into distinct categories. It consisted the flatten layer, two dense layers and a softmax activation layer. The flatten layer converts the multi-dimensional output from the three ECA blocks into a one-dimensional vector, allowing the model to effectively process the feature representations. Following the flatten layer, the first dense layer contains 32 units, applying weights to the flattened input and enabling the model to learn complex patterns and relationships in the data. And the second dense layer, consisting of 4 or 2 units, the number is the number of final classification task, further refines these features, enhancing the model’s ability to differentiate between classes. Finally, the softmax activation layer produces a probability distribution over the output classes, the calculation for the softmax output layer is as follows Equation 5:


(5) $$\hat{y}=\mathrm{softmax}\left(z\right)=\mathrm{softmax}\left(W^{T}x+b\right)$$

where *x* represents the input to the second dense layer, $W^{T}$ denotes the weight matrix, *b* is the bias term, and $\hat{y}$ is the output probability of the softmax function, expressed as Equation 6:


(6) $$\mathrm{softmax}\left(z\right)=\frac{e^{z}}{\sum_{K}e^{z}}$$

where *K* represents the number of labelled outputs. In EEG decoding tasks, *K* = 4 or 2.

The classification block integrates the outputs from three parallel structures and employs two dense layers for hierarchical feature learning. This enhances the expressive power of model, allowing it to capture more complex patterns than a single fully connected layer. As a result, it leads to improved classification performance, achieving better results for MI-EEG decoding.

## 3 Experiment results and discussion

### 3.1 Experimental details and performance metrics

To ensure the validity of all experiments, identical settings were employed across all test cases: all experiments are conducted using the same experimental setup: the AMEEGNet model is built within the Pytorch 1.12 framework using Python 3.10 and trained on an Nvidia GTX 3060 with 12GB of memory. The Adam optimizer is employed to optimize the model parameters, with hyperparameter settings including a learning rate of 0.001, a batch size of 64, and a total of 1,000 epochs using the cross-entropy loss function.

Performance metrics of model is depending on the

(1) Accuracy

(7) $$\mathrm{Accuracy}=\frac{TP+TN}{TP+TN+FP+FN}$$

Equation 7 is formula for accuracy. Where TP represents true positives (correctly identified as positive), FP denotes false positives (incorrectly marked as positive), TN refers to true negatives (correctly identified as negative), and FN indicates false negatives (positive cases incorrectly labeled as negative).

(2) Kappa score

(8) $$\mathrm{Kappa}=\frac{P_{o}-P_{e}}{1-P_{e}}$$

Equation 8 is formula for Kappa score. Where *P*_o_ is the overall accuracy rate, *P*_e_ is the random classification accuracy rate. Additionally, to statistically verify the significant differences between AMEEGNet and other comparison networks, the Wilcoxon signed-rank test (Ohyver et al., 2019) is employed to analyze the network results and conduct significance testing.

### 3.2 Within-subject results of proposed methods

This section aims to evaluate the performance of AMEEGNet by comparing it against established baseline methods. The performance evaluation is conducted using the BCI IV 2a, 2b and HGD datasets. The baseline methods include one traditional machine learning method and six state-of-the-art deep learning methods, described as follows: Antony et al. (2022) utilized online recursive independent component analysis to analyze seven principal components and employed adaptive SVM for classification. Lawhern et al. (2018) introduced EEGNet, which leverages one-dimensional and deep convolutional layers for real-time feature extraction. Hermosilla et al. (2021) proposed ShallowConNet, which utilizes two layers of small convolutional kernels, offering advantages in fast decoding and ease of training. Chen et al. (2024) proposed EEGNeX, which employs 8 and 32 filters in CNNs to achieve a lightweight decoding architecture. Altuwaijri et al. (2022) developed MBEEGNet and MBEEGSE, incorporating multi-branch CNNs and squeeze-and-excitation (SE) attention blocks. Musallam et al. (2021) utilized temporal convolutional networks (TCN) with residual blocks to optimize the architecture.

Table 1 present the within-subject results of proposed and baseline method on the BCI IV 2a, 2b and HGD datasets. The Wilcoxon signed-rank test is employed to statistically analyze the differences between the baseline and the proposed network on BCI 2a. The *p*-values presented in this section are derived from the analysis of the proposed network. When compared with one traditional machine learning method and six state-of-the-art deep learning methods, the machine learning approach achieves a *p*-value of *p* < 0.001. This indicates that the proposed model demonstrates significant improvements over the traditional machine learning approach. And all deep learning approaches demonstrate superior performance in both the two-class (2b dataset) and four-class (2a dataset, HGD) decoding tasks. This indicates that traditional machine learning methods struggle with nonlinear features, resulting in a significant difference when compared to the proposed network, and deep learning methods are effective at extracting complex features from MI-EEG data. While EEGNet (*p* < 0.02), EEGNeX (*p* < 0.001), and ShallowConvNet (*p* < 0.001) achieve commendable accuracy and *k*-scores, TCN (*p* < 0.2) excels in extracting temporal-spatial features, resulting in improved performance. However, the proposed AMEEGNet, with its multi-scale feature extraction, surpasses TCN by 2.41% accuracy and 0.032 *k*-score in the 2a dataset, by 3.85% and 0.077 in the 2b dataset, and 2.77% and 0.0369 in the HGD. Both MBEEGNet (*p* < 0.001) and MBEEGSE (*p* < 0.2) utilize multi-scale extraction architectures, with MBEEGSE benefiting from squeeze-and-excitation (SE) attention, thus achieving better performance. With fusion transmission mechanism, AMEEGNet facilitates more comprehensive feature learning and extraction. Coupled with its lightweight ECA block, AMEEGNet surpasses MBEEGSE by 3.72% and 0.050 in the 2a dataset, by 0.61% and 0.013 in the 2b dataset, and by 2.1% and 0.028 in the HGD. In addition, the proposed method have relatively small standard, it shows that the proposed method has better decoding stability. In terms of *p*-value results, AMEEGNet shows superior improvement effects among the deep learning methods.

**Table 1:** Within-subject experiment results on the three datasets.

Method	BCI IV 2a			BCI IV 2b			HGD		
	Accuracy	Standard	*k*-score	Accuracy	Standard	*k*-score	Accuracy	Standard	*k*-score
CSP + SVM (Antony et al., 2022)	66.44	10.16	0.538	78.72	12.48	0.667	87.72	7.02	0.8380
EEGNet (Lawhern et al., 2018)	77.12	12.86	0.702	86.55	9.77	0.744	92.99	5.93	0.9065
ShallowConvNet (Hermosilla et al., 2021)	75.28	9.83	0.661	86.60	**7.96**	0.732	92.72	6.74	0.9029
EEGNeX (Chen et al., 2024)	74.54	12.55	0.672	83.70	9.10	0.674	87.04	6.32	0.8273
MBEEGNet (Altuwaijri et al., 2022)	76.12	9.12	0.669	89.21	8.21	0.784	93.83	4.28	0.9178
MBEEGSE (Altuwaijri et al., 2022)	77.45	11.61	0.699	89.22	8.34	0.784	93.39	6.04	0.9118
TCNet (Musallam et al., 2021)	78.76	**7.38**	0.717	85.98	9.17	0.720	92.72	5.16	0.9029
Proposed	**81.17**	10.43	**0.749**	**89.83**	8.07	**0.797**	**95.49**	**4.08**	**0.9398**

Bold values represent the best results.

From Table 1, it is evident that AMEEGNet achieves the highest accuracy and *k*-scores for both the two-class and four-class decoding tasks, demonstrating its effectiveness and robustness in MI-EEG decoding.

**Figure 4:**
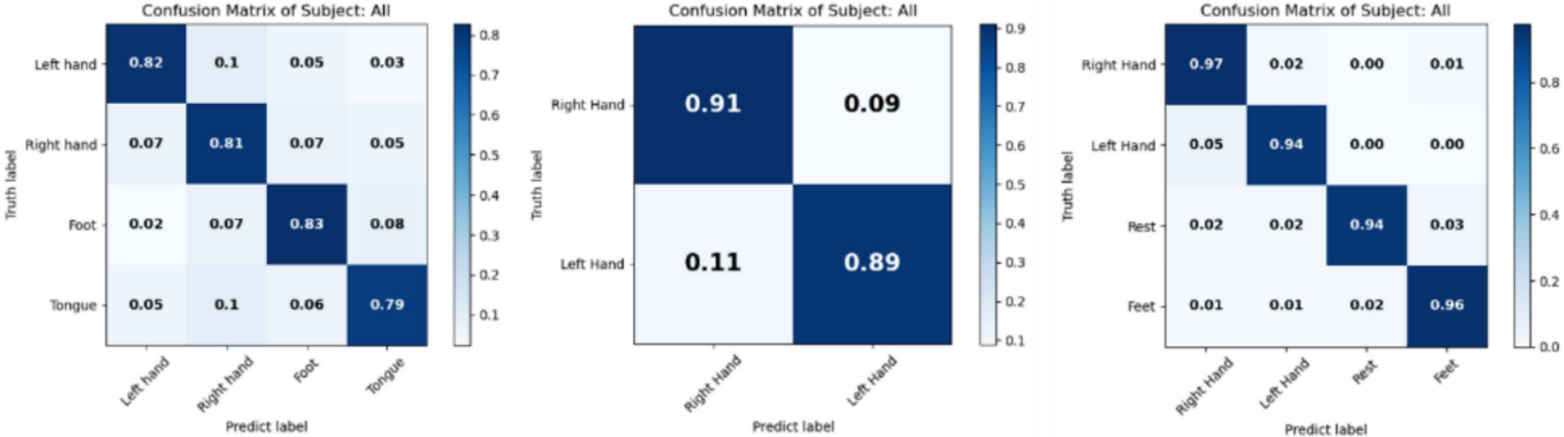
Confusion matrix of results on BCI IV 2a, 2b, and HGD. Left is for 2a, middle is for 2b and right is for HGD.

To evaluate the performance of the proposed method across different classification tasks and identify which tasks are easily confused, the confusion matrices for the BCI IV 2a, 2b and HGD datasets are presented in Figure 4. The left matrix shows high accuracy for the left hand, right hand, and foot classes, indicating effective discrimination among these tasks. However, the tongue class has an accuracy below 0.8, suggesting potential overlap with other classes. The middle matrix illustrates excellent classification capabilities in the two class task, with high diagonal values indicating effective distinction between the two classes. The right matrix illustrates comprehensive decoding performance across four classes. Overall, the proposed method demonstrates strong decoding performance and classification ability across all three datasets.

### 3.3 Cross-independent results of proposed methods

This section focuses on assessing cross-subject decoding performance through a subject-independent experiment. The Leave-One-Subject-Out (LOSO) evaluation method designates one subject from the dataset as the test set, while the remaining subjects are used to create the training set. This approach effectively evaluates the cross-subject decoding performance of model. Table 2 presents the results of the LOSO experiment on the BCI 2a dataset, using accuracy, standard deviation, and Kappa score to assess the decoding performance of each model. The proposed method achieves the highest accuracy of 66.68% and a Kappa score of 0.5558, indicating strong decoding performance for previously unseen subjects and robust applicability in real-world scenarios.

**Table 2:** Leave-One-Subject-Out (LOSO) experiment results on the BCI 2a datasets.

Method	S01	S02	S03	S04	S05	S06	S07	S08	S09	Accuracy	Standard	*k*-score
CSP + SVM (Antony et al., 2022)	50.00	53.43	62.18	44.59	55.21	50.65	46.87	58.03	42.22	51.46	6.08	0.3564
EEGNet (Lawhern et al., 2018)	68.75	53.12	77.08	60.59	55.21	52.95	73.44	78.99	66.32	65.16	9.60	0.5355
ShallowConvNet (Hermosilla et al., 2021)	62.57	51.04	80.21	60.07	53.65	51.56	72.74	82.64	72.22	65.18	11.49	0.5507
EEGNeX (Chen et al., 2024)	61.63	58.68	67.19	59.72	68.92	60.94	75.00	64.41	61.98	64.27	**4.95**	0.5237
MBEEGNet (Altuwaijri et al., 2022)	67.53	50.00	83.16	62.50	56.77	51.56	73.61	82.81	68.75	66.30	11.58	0.5508
MBEEGSE (Altuwaijri et al., 2022)	67.53	49.31	81.6	61.81	53.82	48.61	71.18	83.33	69.79	65.22	12.15	0.5363
TCNet (Musallam et al., 2021)	62.5	50.00	81.08	58.68	49.61	49.31	70.83	73.44	68.06	62.61	10.93	0.4887
Proposed	69.27	49.83	81.77	61.46	59.72	53.99	72.22	81.77	70.14	**66.68**	10.67	**0.5558**

Bold values represent the best results.

Specifically, compared to the traditional machine learning method CSP + SVM (*p* < 0.02), the proposed method shows a significant improvement of 15.22%. When compared to EEGNet (*p* < 0.2), EEGNeX (*p* < 0.2), and TCNet (*p* < 0.01), the proposed method demonstrates enhancements of 1.52, 2.41, and 4.07%, respectively, highlighting the effectiveness of the multi-scale architecture in improving subject-independent decoding performance. ShallowConvNet (p < 0.2) exhibits similar performance to EEGNet. while the proposed method outperforms the multi-scale deep learning methods MBEEGNet (*p* < 0.2) and MBEEGSE (*p* < 0.2) by 0.38 and 1.46%, respectively. This improvement can be attributed to the integration of fusion transmission and the ECA block, which enhances the decoding capabilities of model. Furthermore, the Wilcoxon signed-rank test indicates that the proposed method exhibits superior improvement effects among the decoding techniques, demonstrating excellent performance for both subject-dependent and subject-independent decoding tasks.

### 3.4 Evaluation of the proposed multi-scale learning strategy

AMEEGNet utilizes several designed blocks to enhance its performance. To evaluate of the proposed multi-scale learning strategy, an ablation study was conducted. Three structures were examined in this study: the parallel EEGNet structure, the fusion transmission structure, and the ECA block structure. The model without any of these structures is referred to as EEGNet, which is analyzed in section 3.2; therefore, this ablation experiment does not include the model without these structures.

Table 3 illustrates the model structures for experiments 1 to 4 on the BCI IV 2a dataset, where “✓” indicates the presence of a structure and “⨯” indicates its absence. Experiment 4 represents AMEEGNet. The results demonstrate that each structure contributes to the decoding effectiveness of model. The model utilizing both the parallel EEGNet and fusion transmission structures performs worse than the one with only the parallel EEGNet, indicating that complex features may not be effectively classified by the classification block alone. However, the inclusion of the ECA block clarifies the features, leading to significant improvements in performance of AMEEGNet.

**Table 3:** Ablation experiment results of AMEEGNet on BCI IV 2a.

Exp.	Parallel EEGNet	Fusion transmission	ECA block	Accuracy	Standard	*k*-score
1	✓	⨯	⨯	78.32	9.85	0.715
2	✓	✓	⨯	79.40	10.40	0.725
3	✓	⨯	✓	79.01	11.22	0.720
4	✓	✓	✓	81.17	10.43	0.749

**Figure 5:**
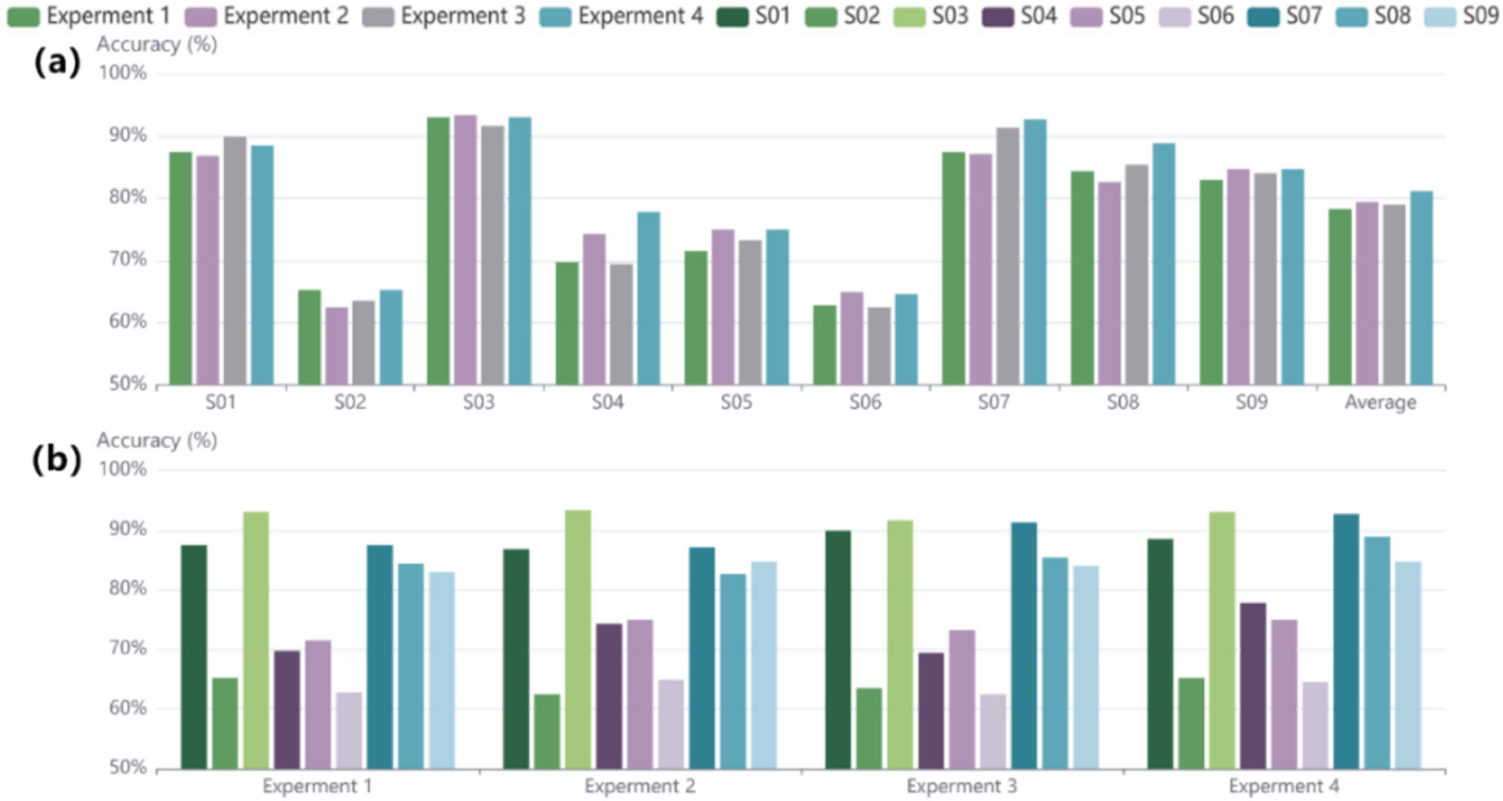
Results of the ablation experiments conducted on subjects in the BCI IV 2a dataset. **(A)** Displays the performance results of the ablation experiments for each individual subject. **(B)** Shows the overall performance outcomes for each ablation experiment across subjects.

To more accurately assess the impact of the ablation experiments on each subject, the accuracy of every subject based on the BCI 2a dataset was also examined. In Figure 5A, the results indicate significant improvements for subjects S04, S07, and S08, suggesting that the designed structure enhances the stability of decoding performance across different individuals. Figure 5B shows that experiments 1, 2, and 3 exhibit similar trends; however, the improvement in decoding effectiveness is clearly evident in experiment 4. This indicates that the use of structures alone is not sufficient, whereas the combination of structures yields better feature extraction results, highlighting the effectiveness of the structural design and their combined application. To verify and analyze the results from a statistical perspective, the Wilcoxon signed-rank test is employed. The results of experiment 4 showed significant differences compared to experiments 1, 2, and 3, with *p*-values of *p* < 0.05, *p* < 0.02, and *p* < 0.001. These findings highlight the importance of the proposed enhancements, which improve accuracy and provide a statistically significant advantage over earlier methodologies.

### 3.5 Visualization

**Figure 6:**
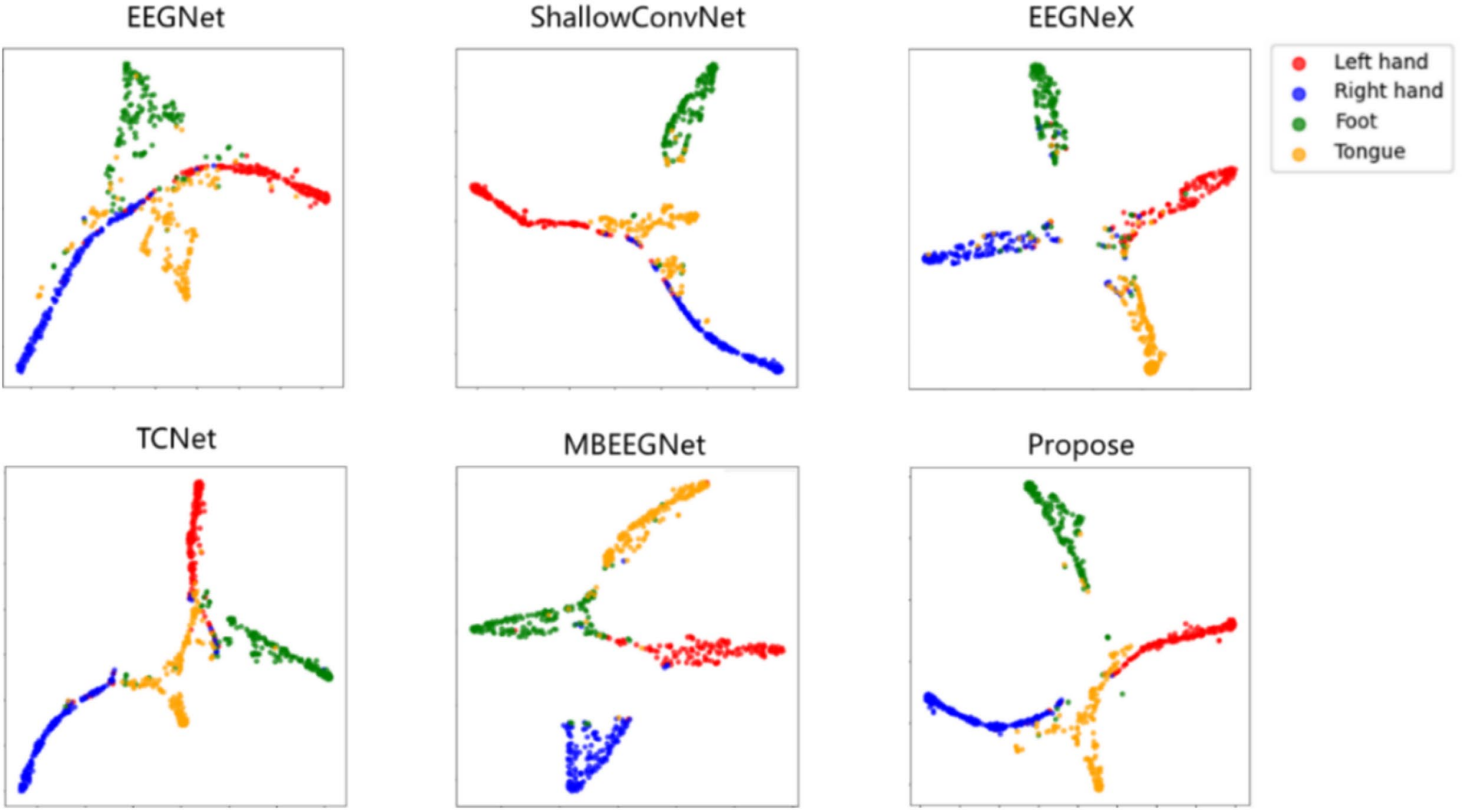
T-SNE visualization of deep learning methods.

To visually evaluate the proposed AMEEGNet and baseline methods and demonstrate the effectiveness of channel attention, visualization techniques were employed. Figure 6 presents the *t*-distributed stochastic neighbor embedding (Svantesson et al., 2023) (T-SNE) visualization results of various deep learning methods. The results show significant overlap in the classification tasks for EEGNet, TCNet, and MBEEGNet. Different colors represent the various classification tasks; the left and right hand classifications show clear separation across all networks, in contrast, the tongue class task of ShallowConvNet exhibits considerable dispersion, leading to poor classification performance. EEGNeX displays relatively clear classification boundaries but suffers from numerous misclassifications across all four categories. The proposed model demonstrates a more concentrated classification effect for each task, although the tongue class task remains somewhat diffuse. Future research could focus on improving classification performance in the tongue task, as well as enhancing the overall MI-EEG decoding effectiveness.

To examine the spatial features learned by proposed model, Gradient-weighted Class Activation Mapping (Grad-CAM) (Li et al., 2020) is utilized to emphasize EEG signals based on these features, visualizing the activated spatial components during MI EEG decoding on EEG topography maps.

**Figure 7:**
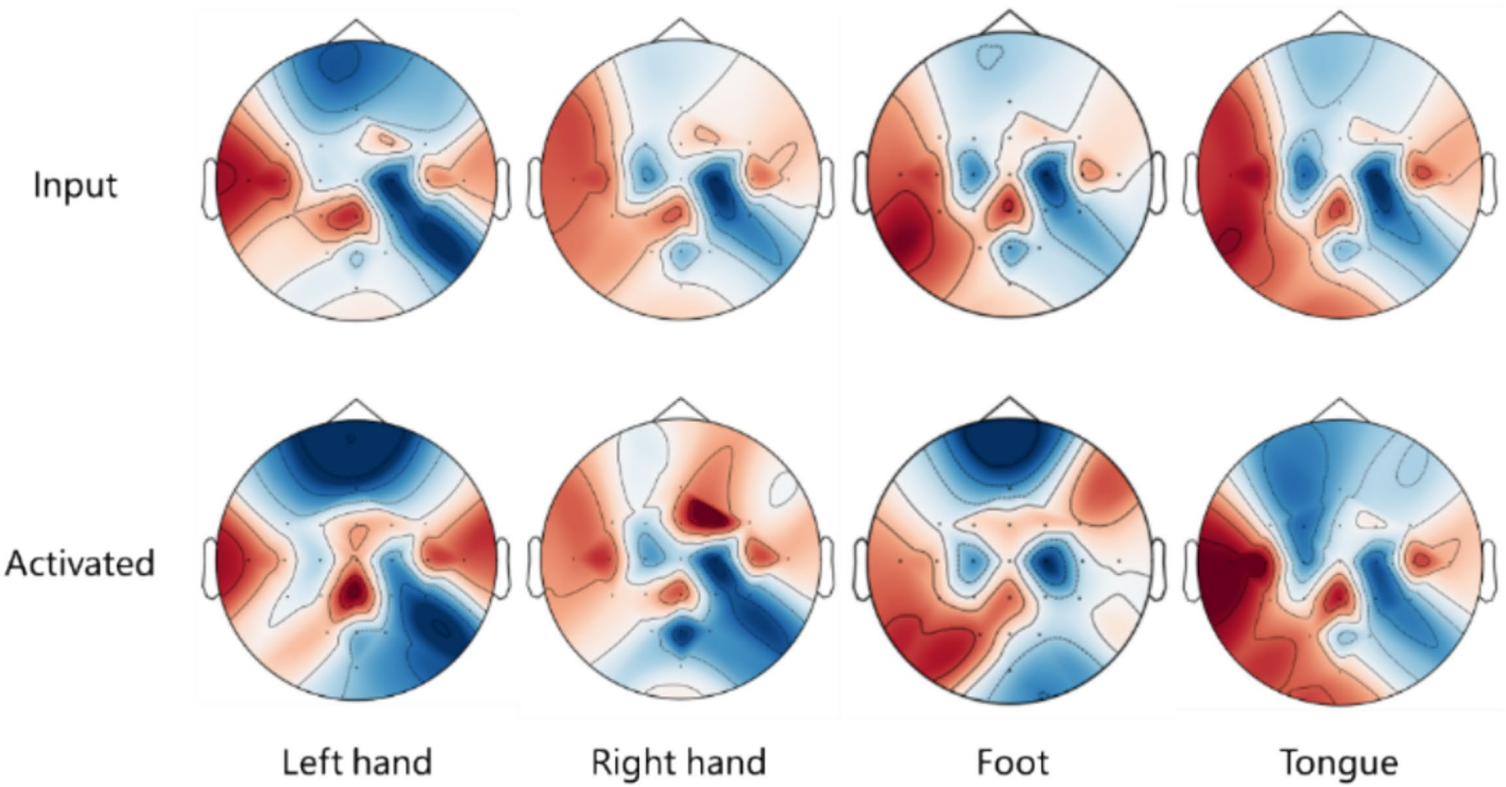
EEG topographic heat map comparison between input signals and activated signals in BCI IV 2a dataset.

Figure 7 presents a comparison of the EEG topographic heat maps between input signals and activated signals from the BCI IV 2a dataset. The upper part of the figure illustrates the influence of the input EEG signals on the final classification task, with red regions indicating a stronger impact on classification and blue regions representing a weaker effect. This visual representation helps identify which areas of the input EEG signals are most significant for the model’s predictions. The lower part of the figure shows the heat map activated during the decoding process. Here, red regions signify areas of the network that are fully utilized for activation during decoding, while blue regions indicate areas that are less effectively activated. By comparing the upper and lower parts of the figure, it is possible to determine whether the model activates and utilizes important signals. Overlapping red and blue areas indicate that the model effectively uses significant signals while disregarding those that are less important.

Notably, different classification tasks exhibit distinct activated regions, indicating that the model concentrates on specific features relevant to each task. In each classification task, there is a significant overlap of red and blue areas, suggesting that the model effectively identifies and activates important spatial features for decoding MI EEG tasks. For the tongue task, the activated regions resemble those of the left and right hand tasks, which may account for the unclear classification performance observed in the T-SNE results. This overlap underscores the difficulty of distinguishing the tongue class from the other tasks, indicating that classifying tongue tasks from a spatial perspective presents challenges. Future efforts could focus on enhancing the decoding performance of tasks, which currently suffer from poor spatial resolution, by exploring other dimensions, such as temporal aspects, to improve the overall performance of the model.

## 4 Discussion and conclusion

To improve the power of model extract more distinguishable temporal-spatial features, enhance the effect of MI-EEG decoding, thereby pushing the BCI development, this study proposes an attention-based multiscale EEGNet (AMEEGNet), the model exhibits excellent performance in MI-EEG decoding. First, AMEEGNet use three parallel EEGNet to achieve the effect of multiscale feature extraction, addition of fusion transmission method, achieve the larger degree effect of multiscale feature extraction. Then, the ECA block is used to flexibly capture the dependency relationships between channels, giving greater weight to important channels, improve the power of model feature extraction. Finally, a fallen layer integrates the output of three parallel network, and decoding signals with two dense layers and softmax activation function.

Some comparative experiments were conducted based on publicly datasets, On the BCI Competition IV 2a dataset, an average classification accuracy of 81.17% and an average classification kappa value of 0.749 were achieved. Its four class classification accuracy and kappa value were significantly higher than traditional machine learning algorithms and currently more advanced deep learning algorithms. In addition, on the BCI Competition IV 2b dataset, the proposed method achieved an average classification accuracy of 89.83% and an average classification kappa value of 0.797, demonstrating outstanding decoding advantages over the compared network.

Future work will focus on further exploring the temporal aspects of the model to enhance performance in dynamic environments. Specifically, we aim to integrate recurrent neural networks (RNNs) to capture contextual relationships within the proposed network. This may involve combining AMEEGNet with long short-term memory (LSTM) networks for parameter tuning, which could improve the decoding performance of motor imagery (MI) EEG signals.

In addition, future research will also address the challenges of online decoding, emphasizing the importance of short-time decoding strategies. By focusing on real-time processing, aiming to enhance the model’s responsiveness and accuracy in practical applications, paving the way for more effective BCI.

## Data Availability

Publicly available datasets were analyzed in this study. This data can be found here: https://www.bbci.de/competition/iv/#download.
